# Expression of Stem Cell Markers in Preinvasive Tubal Lesions of Ovarian Carcinoma

**DOI:** 10.1155/2015/808531

**Published:** 2015-10-04

**Authors:** G. Chene, V. Ouellet, K. Rahimi, V. Barres, L. Meunier, M. De Ladurantaye, D. Provencher, A. M. Mes-Masson

**Affiliations:** ^1^Centre de Recherche du Centre Hospitalier de l'Université de Montréal, Montreal, QC, Canada H2X 0A9; ^2^Institut du Cancer de Montréal, Montreal, QC, Canada H2X 0A9; ^3^Department of Gynecology, Hôpital Femme Mère Enfant, Hospices Civils de Lyon, 69677 Bron, France; ^4^Université Claude Bernard Lyon 1, EMR 3738, 69000 Lyon, France; ^5^Department of Pathology, Centre Hospitalier de l'Université de Montréal, Université de Montréal, Montreal, QC, Canada H2X 0A9; ^6^Division of Gynecologic Oncology, Université de Montréal, Montreal, QC, Canada H2X 0A9; ^7^Department of Medicine, Université de Montréal, Montreal, QC, Canada H2X 0A9

## Abstract

In order to better understand the ovarian serous carcinogenic process with tubal origin, we investigated the expression of stem cell markers in premalignant tubal lesions (serous tubal intraepithelial carcinoma or STIC). We found an increased stem cell marker density in the normal fallopian tube followed by a high CD117 and a low ALDH and CD44 expression in STICs raising the question of the role of the stem cell markers in the serous carcinogenic process.

## 1. Introduction

The model of cancer genesis from stem cells is based on the principle of self-renewal of these cells, followed by their differentiation into multipotent progenitors and finally an evolution towards differentiated cancer cells. This population of cancer stem cells, probably representing less than 5% of all tumor cells, would be responsible for the degree of aggressiveness of the disease, the metastatic potential, and chemoresistance [1, 2].

Various ovarian cancer stem cell markers have been described, such as ALDH1, CD44, and CD117. ALDH1 (also known as ALDH1A1) is an enzyme involved in the metabolism of retinoic acids and probably plays a central role in cellular differentiation [3]. The retinoic acid system is involved in chromosome stability and epigenetic regulation and is probably a protective mechanism against alterations in stem cells related to oxidative stress [3]. ALDH1 is also involved in the modulation of various signalling pathways (e.g., AKT/*β*-catenin, WNT, and p21-p53…) which are in turn involved in the molecular regulation of cancer stem cells.

CD44 is a cell-surface glycoprotein involved in invasion and metastasis via the activation of the PI3K/AKT pathway [4, 5]. CD117 is a protooncogene (c-kit) that encodes for a tyrosine kinase receptor and plays an important role in oncogenic process such as cell proliferation and tumor development [6].

An ovarian serous carcinogenic sequence was recently described and it has been suggested that most high-grade serous ovarian cancers (HGSC) would have a tubal origin and a tubal precursor lesion called “serous tubal intraepithelial carcinomas (STICs)” could metastasize to the ovary and adjacent peritoneum [7]. We have previously demonstrated that there was an activation of the DNA damage response machinery in STICs which could consequently trigger the invasive carcinogenic process [8, 9]. Of note, little is known about the stem cell profile of STICs. A recent study has demonstrated that the loss of ALDH1A1 is associated with tumor progression from STIC to HGSC [10]. In order to further validate this finding, we have included additional stem cell-associated markers in a cohort of STICs and HGSC in order to describe their expression along the neoplastic continuum.

## 2. Material and Method

### 2.1. Patients and Clinical Data

Tissue samples were obtained from patients who underwent surgery between 1993 and 2012 for either prophylactic salpingo-oophorectomy or ovarian cancer at the Division of Gynecologic Oncology (Centre hospitalier de l'Université de Montréal (CHUM), Hôpital Notre-Dame). All patients gave consent for the banking and use of their tissue samples and clinical data (SARDO database). The ethic review board at the CHUM approved the study.

### 2.2. Tissue Microarray (TMA) Construction

A pathologist specialised in gynecologic oncology reviewed all cases. STICs were defined by presence of nonciliated cells exhibiting high immunohistochemical expression of TP53 and Ki67 and the presence of 3 or more of the following features: abnormal chromatin pattern, nuclear enlargement, marked nuclear pleomorphism, epithelial stratification, and/or loss of polarity or nuclear molding [7]. Areas of interest were circled on the H&E section and a representative core (0.6 mm) of each specimen was arrayed on a receiver paraffin block using the MArrayer (Pathology Devices). Due to the small size of STIC lesions, only one core was arrayed on the TMA. TMAs were cut at 5 *μ*m thickness and sections were laid onto Superfrost + glass slides. Once completed, a TMA section was stained with hematoxylin-eosin to receive a final pathology review.

The TMA included 21 benign-appearing fallopian tubes, 21 STICs (from the same patients as the benign-appearing fallopian tubes), 17 HGSC from patients with STICs (associated ovarian cancer or AOC), and 30 HGSC without STICs (non-AOC). Only chemonaive cases from patients without any BRCA germline mutation were considered for this study.

### 2.3. Western Blot

The specificity of each antibody was assessed by Western blot using PC3, DU145, LNCaP, OVCAR 3 cell lines, benign prostatic (RWPE), and benign ovarian cells (BOV 2655G and BOV 2567D). Antibody conditions were defined using an optimisation TMA containing cell pellets of cancer cell lines from several origins including prostate (LNCaP, DU145, and PC3), ovary (OV90, SKOV3, TOV1946, and TOV81D), and breast (MCF-7), in addition to benign prostatic cells (RWPE), Hela cells (irradiated and nonirradiated cells), Jurkat cells, and benign tubal cores.

### 2.4. Immunohistochemistry

Staining of all antibodies was performed using the Benchmark XT autostainer (Ventana Medical System Inc.). Antigen retrieval was performed with Cell Conditioning 1 (Ventana Medical System Inc., number 950-124) during 30 or 60 minutes for most antibodies although the Cell Conditioning 2 (Ventana Medical System Inc., number 950-124) was used for TP53. Prediluted antibodies were manually added to the slides and incubated at 37°C for 20 to 60 minutes. The following antibodies and dilutions were used: Ki67 (1 : 500; Clone SP6, RM-9106, NeoMarkers), p53 (1 : 200; Clone DO-1, sc-126, Santa Cruz Biotechnologies), ALDH1 (1 : 400, Clone 44/ALDH, 611194, BD transduction lab), CD44 (1 : 100, Clone 2F10, BBA13, RD System), and CD117 (1 : 200, c-kit, Dako). Staining was revealed using the UltraView universal DAB detection kit (Ventana Medical System Inc., 760-500). Counterstaining was achieved with hematoxylin and bluing reagent (Ventana Medical System Inc., number 760-2021 and number 760-2037). Substitution of the primary antibody with phosphate-buffered saline served as a negative control. All sections were scanned using a VS-110 microscope with a 20x 0.75NA objective with a resolution of 0.3225 *μ*m (Olympus). Images were analysed with the OlyVIA software (Olympus).

### 2.5. Scoring and Statistical Analysis

Protein expression was scored according to the extent (as a percentage of cells of interest) and intensity (value of 0 for absent, 1 for low, 2 for moderate, and 3 for high) of staining based on no-automated visualization and a previously described semiquantitative score was used [11]. All slides were independently scored in a blind manner by 2 observers and interrating agreement was >80%. In case of differences between the two scorings, the core was reevaluated to reach a consensus. Statistical analyses were performed using SPSS Statistics 20 software (IBM). The nonparametric Mann-Whitney *U* test was used to compare protein expression between groups. A *p* value below 0.05 was considered as statistically significant.

## 3. Results

As expected, CD117 and CD44 expression were cytoplasmic and membranous in epithelial tissues. Cytoplasmic staining was seen for ALDH1 protein in both epithelium and stroma. The uniform ALDH1 expression in stromal cells constituted an internal control. TP53 and Ki67 expression were nuclear and were used to confirm the diagnosis of STICs. Briefly, STICs were characterized by an intense and diffuse expression of TP53 with a moderate to high Ki67 proliferative index. Expression of TP53 and Ki67 was absent in benign-appearing fallopian tubes whereas it was intense and higher in AOC and non-AOC.

**Figure 1:**
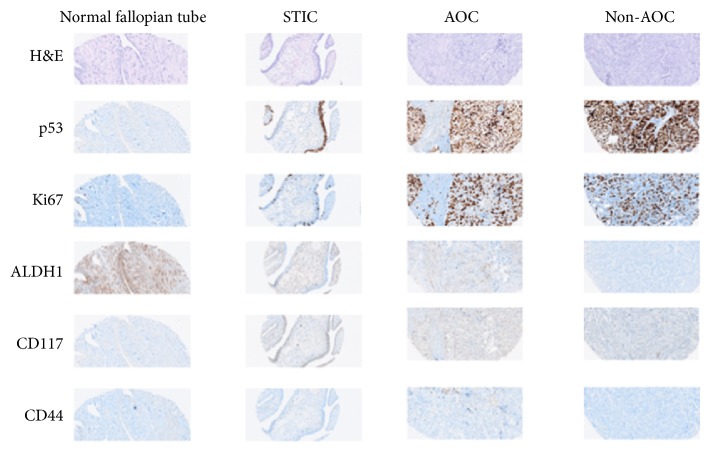
Immunoprofiles of p53, Ki67, ALDH1, CD44, and CD117 in normal-appearing fallopian tube, STIC, AOC (associated carcinoma with STICs), and non-AOC (carcinoma without STICs). STICs are defined by an intense and diffuse expression of TP53 with a moderate to high Ki67 proliferative index. Expression of TP53 and Ki67 is absent in benign-appearing fallopian tubes whereas it is intense and higher in AOC and non-AOC. ALDH1A and CD117 staining is detected in both secretory and ciliated cells of the benign-appearing fallopian tubes. The level of ALDH1 expression is significantly lower in STICs, AOC, and non-AOC than in normal fallopian tube. CD117 expression level is as high as in benign tube and AOC but significantly lower in non-AOC. There is also an increase in the CD117 positive cells in the STICs compared to fallopian tube epithelium. The expression level of CD44 was weak or absent in most specimens. However, expression is significantly lower in non-AOC than in benign tubes, STICs, and AOC.

**Figure 2:**
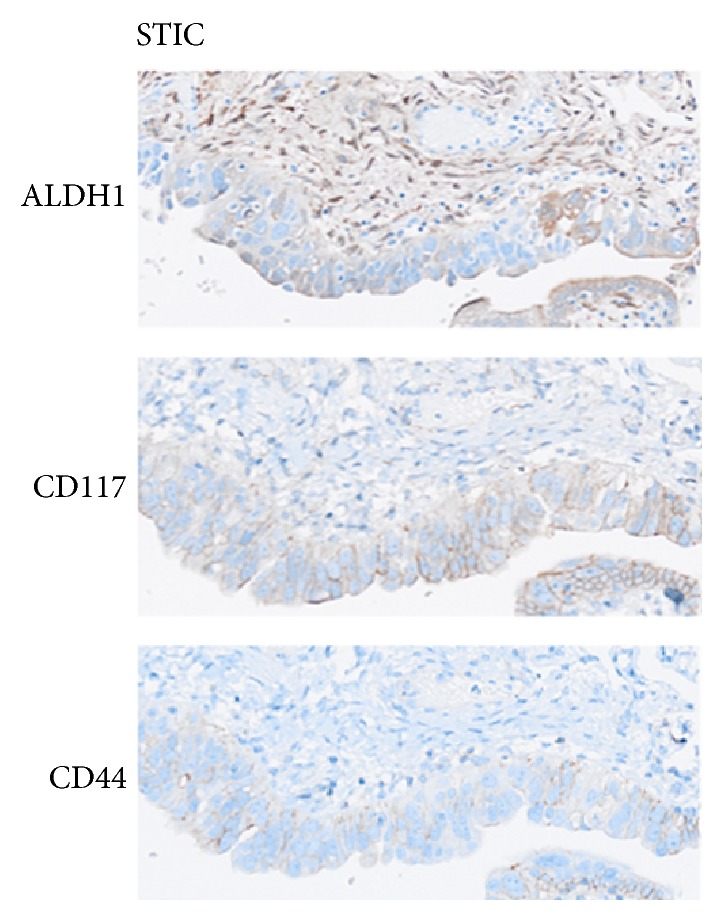
ALDH1, CD117, and CD44 overexpression in some cells of a STIC lesion, suggesting a possible and specific location for stem cells within these lesions and a possible stem cell niche.

ALDH1A staining was detected in both secretory and ciliated cells of all the 21 benign-appearing fallopian tubes (long and discontinuous stretches of high immunoreactivity) whereas the level of expression of ALDH1 was significantly lower in STICs, AOC, and non-AOC (*p* = 0.001) (see Table 1 and Figure 1). Interestingly, there were rare positive cells with high ALDH1 expression in 3 STICs; there was also a CD117 and CD44 overexpression in these same cells (see Figure 2). CD117 expression level was as high as in benign tube (located in both secretory and ciliated cells) and AOC but significantly lower in non-AOC (*p* = 0.01). While there appeared to be an increase in the CD117 positive cells in the STICs compared to fallopian tube epithelium, this difference was not statistically significant (Table 1).

The expression level of CD44 was weak or absent in most specimens. However, expression was significantly lower in non-AOC as compared to benign tubes, STICs, and AOC (*p* = 0.001).

Finally, differences in immunohistochemical profiles between AOC and non-AOC were also noted (Table 1).

## 4. Discussion

The recent establishment of STICs as precursor lesions for HGSC opens up new horizons in the understanding of ovarian carcinogenesis and could lead to the development of new screening and/or early prevention strategies [7]. Historically, the origin of ovarian cancer was thought to be ovarian from the ovarian surface epithelium. This was followed by a paradigm shift with the establishment of a tubal origin of most HGSC. Indeed, these cancers may originate in the epithelium of the fimbriae. A lesion named STICs (located almost always in the fimbriae) has recently been reported in prophylactic salpingo-oophorectomies for BRCA mutation as well as in the tubal fimbriae from women with sporadic nonhereditary HGSC. There is a molecular lesional continuity between STICs and ovarian cancer with identical mutations of TP53 [9, 12]. STICs are also characterised by an important genetic instability as shown by the overexpression of the marker of double-strand DNA breaks *γ*H2AX [9]. In this new serous carcinogenic sequence, there may be metastasis exfoliation from STICs to the adjacent ovary and the peritoneum [12]. Little is known about the molecular pathways of STICs.

Regulation of noncancerous stem cells is a complex balance between cellular proliferation, cellular differentiation, and cell death via various signaling pathways including Sonic Hedgehog Shh, Notch, and Wnt. If a deregulation of these signalling pathways is associated with certain mutations, this could result in carcinogenesis due to the appearance of cancer stem and progenitor cells. Deregulation in these signalling pathways is associated with mutations providing the affected cell with stem/progenitor cells characteristics leading to carcinogenesis. Such mutations represent one of the major alterations that might explain the different histological types found in ovarian cancers. Indeed, cancer stem cells and progenitor cells bearing p53 mutations and BRCA (BRCA mutations in genetic cases or BRCA functional abnormalities in sporadic cases) would result in serous tumors and those with *β*-catenin and PTEN would lead to endometrioid tumors. Less is known in the mutations leading to mucinous and clear cell histotypes but p53 seems to be a trigger [2].

The parallel with breast cancer could be suggestive because of the epidemiological and molecular relationship with the ovarian cancer. This type of association was also found in breast cancer where experimental and* in vitro* works demonstrate that BRCA1 is a mammary stem and progenitor cells regulator, allowing their differentiation into mature luminal and myoepithelial cells [13, 14]. BRCA1 mutations would result in overexpression of ALDH1 at mammary cell level and would prevent differentiation of these stem cells [14]. Since BRCA1 is also involved in DNA repair and genomic stability, it has been proposed that BRCA mutations or functional abnormalities would result in accumulation of genetically unstable mammary stem cells and thus a step towards breast cancer [15, 16].

The highest ALDH1 expression is found in the normal-appearing fallopian tube in our study. Auersperg [17] studied the stem cell profile of oviductal fimbriae with 5 stem cell markers (NANOG, SFRP1, LHX9, ALDH1A1, and ALDH1A2); the author found that the fimbriae express the stem cell markers (mainly ALDH1A1, also known as ALDH1) and concluded that the fallopian tube may be pluripotent with the capacity to generate cancer stem cells. Similarly, another study found that stemlike cells (epithelial cell adhesion molecule, CD44, and integrin *α*6) are concentrated in the fimbriated distal end of the tube [18]. The results indicate that STICs may express CD44 and KRT 5 suggesting that these cells may play a role in the initiation of HGSC. However, while we agree with the probable biological adaptation for repair (due to genotoxic stress during ovulation) in the normal fallopian tube, we and others [10] did not find an increase in expression of all the stem cells in STICs: ALDH1 and CD44 expressions were low in STICs whereas we found a more elevated CD117 expression in STICs compared with the normal-appearing fallopian tube (despite the absence of a statistical difference).

Our results are in line with those of Chui et al. [10] where high expression of ALDH1 was noted in normal tubal epithelial cells (29 cases), and then there was an absence of expression in STICs (17 cases). The authors concluded that loss of ALDH1 expression may be an early event in ovarian carcinogenesis which is subsequently turned off later in the process [19]. However, we found some cases with rare positive cells with high ALDH1, CD117, and CD44 expression in STICs, suggesting a possible and specific location for stem cells within these lesions and a possible stem cell niche.

In the other cases where ALDH1 expression is low in STICs, the explanation could be that ALDH1 is not directly involved in the development of STICs. On the contrary, the high expression of CD117 in STICs could mean that CD117 is specifically involved in the pathogenesis and the development of cancer stem cells in STICs. Because kinase inhibitors (such as imatinib mesylate, sunitinib, nilotinib, or dasatinib) target the CD117 positive tumors, other studies are still needed to explore the stem cell profile of the serous tubal carcinogenic sequence [6].

Finally, we have found some differences in the patterns of ALDH1, CD44, and CD117 staining between AOC and non-AOC. In our previous immunohistochemical study on DNA damage signalling and apoptosis in preinvasive tubal lesions, we also highlighted differences between AOC and non-AOC. We hypothesize that AOC and non-AOC may not represent the same lesions. Indeed, these results are intriguing as it can be argument that HGSC could have a dual origin (tubal or ovarian origin) that represents with distinct molecular profiles [9].

## 5. Conclusions

There is emerging evidence that most of HGSC arise from the fallopian tube, especially those associated with STIC lesions [7]. The clinical and pathological significance of stem cells in STICs remains unresolved. It seems that the increased stem cell marker density in the normal fallopian tube may contribute to the carcinogenic process resulting in a low ALDH and CD44 expression in STICs. There could be also an on-off effect of ALDH1 expression during the cancer disease process [19]. Our results hint at the presence of a specific stem cell niche in STICs. In clinical point of view, the higher CD117 expression and the AHDH1 extinction in STICs could be of interest as specific targets [19, 20].

## Figures and Tables

**Table 1:** Protein expression of ALDH1, CD44, and CD117 in the epithelial compartment of normal-appearing fallopian tubes, STICs, AOC (associated carcinoma with STICs), and non-AOC (ovarian carcinoma without STICs).

	Normal-appearing fallopian tube (*n* = 21)	STICs (*n* = 21)	AOC (*n* = 17)	Non-AOC (*n* = 30)
ALDH1 mean scores ± St error	47 ± 7.42	10.37 ± 5.01	7.41 ± 3.02	1.93 ± 0.88
CD117 mean scores ± St error	48.11 ± 9.33	58.29 ± 9.96	44.27 ± 10.95	16.08 ± 4.47
CD44 mean scores ± St error	23.63 ± 7.52	17.24 ± 8.23	13.33 ± 4.74	1.72 ± 4.19
